# Dose-Response Analysis in the Joint Action of Two Effectors. A New Approach to Simulation, Identification and Modelling of Some Basic Interactions

**DOI:** 10.1371/journal.pone.0061391

**Published:** 2013-04-24

**Authors:** Miguel Anxo Murado García, Miguel Ángel Prieto Lage

**Affiliations:** Instituto de Investigacións Mariñas (IIM-CSIC), Galicia, Spain; University of Oxford, United Kingdom

## Abstract

In systems with several effectors, the results of dose-response (DR) experiments are usually assessed by checking them against two hypotheses: independent action (IA) and concentration addition (CA). Both are useful simplifications, but do not represent the only possible responses, and avoid to a large extent the analysis of the interactions that are possible in the system. In addition, these are often applied in such a way that they produce insufficient descriptions of the problem that raises them, frequent inconclusive cases and doubtful decisions. In this work a generative approach is attempted, starting from some simple mechanisms necessarily underlying the response of an elementary biological entity to an effector agent. A set of simulations is formulated next through an equally simple system of logical rules, and several families of virtual responses are thus generated. These families include typical responses of IA and CA modes of action, other ones not less probable from a physiological point of view, and even other derived from common and expectable forms of interactions. The analysis of these responses enabled, firstly, to relate some phenomenological regularities with some general mechanistic principles, and to detect several causes by which the IA-CA dualism is necessarily ambiguous. Secondly, it allowed identifying different forms of synergy and antagonism that contribute to explain some controversial aspects of these notions. Finally, it led to propose two sets of explicit algebraic equations that describe accurately a wide diversity of possible and realistic responses.

## Introduction

The response of a population of biological entities to the action of an effector is typically sigmoidal and requires for its algebraic description (the dose-response model: DR) an equation with at least three parameters. If the response is altered by a perturbation agent, variations depending on the perturbator concentration must be expected in one or more of these parameters. If two effectors interact, one or more parameters corresponding to the action of each effector will vary, in the description of the joint response, as a function of the concentration of the other one. Although these premises are not much debatable, their practical application has the disadvantage of requiring a solution whose complexity increases in a more than linear way with the number of effectors considered.

This justifies the common use of two simplifications: the IA (independent action) [1] and the CA (concentration addition) [2], [3] hypotheses. Both avoid the mentioned disadvantage by postulating conditions that allow verifiable predictions about the joint response, using the individual DR models without adding new parameters. Next we will discuss the details of these hypotheses; now we will point out only that their formalizations are generally considered as empiric models lacking in mechanistic content, what is not completely true.

DR models are considered empirical (phenomenological, macroscopical) because they describe the sensitivity distribution of an effector in a target population. Although this provides DR models with a statistical basis, ultimately the response depends on processes that take place at the level of the interactions between the effector quanta (ions, atoms, molecules, electric pulses, radiations) and the receptor structures of the biological system, a level that is ignored by the model. However, using a thermodynamic analogy, the (macroscopic) sensitivity distribution can be broken down into the (microscopic) distributions of other elements that are response-determining at a finer resolution level. These elements can be physical structures whose reduction to other simpler ones has no sense (as the number of receptors per biological entity), or more complex physiological limits (as a response threshold), but in any case, they can be linked in biological systems with the effector quanta of an agent through hypotheses about some general forms of molecular interactions.

Under this perspective, IA and CA hypotheses postulate modes of action that can be associated to general mechanisms or microscopic conditions, which allows to propose variations capable of generating specific responses. To classify these variations from bibliographic data is difficult due to: the interference of the experimental error; the required categories are not usually considered in toxicodynamic studies; and the suitable designs for a given hypothesis rarely can be used to prove facts outside of their conceptual framework. In this sense, a way for eluding these difficulties can be achieved by performing simulation “experiments”. Both, the statistical basis and the general types of mechanisms underlying the DR relationships (interactions between cell receptors, effectors and interfering agents) are sufficiently known for simulating microscopic conditions able to produce the corresponding macroscopic (populational) results.

In the simulations used in this work, simple properties for the microscopic determinants of the response were postulated, and a set of basic “sigmoidal scenes”–among them those associated with IA and CA hypotheses– were generated with the only assistance of logical (Boolean) rules. Additionally, more specific response surfaces were obtained by including in such rules some algebraic expressions describing concrete interactions as those that can take place in many physiological contexts (activation/deactivation, competence/cooperation, steric hindrance). The results allowed to illustrate the status of IA and CA hypotheses within the field of the possible responses, to characterize several types of perturbations and interactions, and to propose explicit algebraic models that translate the mechanics of the response into specific parametric variations. Although in some cases the practical utility of these models can be limited by a low number of observations and a high experimental error, the simulations constitute always a useful reference for interpreting a complex response, inferring the type of mechanism involved and suggesting complementary experiments.

## Theoretical Background and Methods

### 1. Numerical methods

Hereafter we will call effector any agent able to cause a (typically sigmoidal) response in a population of biological entities, and perturbator to any agent that can alter the response to an effector, itself being unable to cause it. Receptor is any biological structure acting as ligand of effectors or perturbators. The term dose is reserved to the concentration of an effector.

In the simulation procedures that are described later, the Weibull's random numbers *w*:(θ;α) were obtained, from the uniform random numbers *u*:[0,1] provided by the spreadsheet, through [4], [5]:
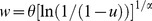
where mean (μ_w_) and standard deviation (σ_w_) of the corresponding distribution are:
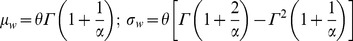



To facilitate comparisons, doses were coded into the [0-(0.1)-1] interval, and responses were calculated by considering *Y* as a total number of biological entities, *S* the survive population at a given dose, therefore the surviving population response *R* can be expressed in a useful coded range [0,1] as *R* = 1−(*S*/*Y*). The simulated and experimental results were adjusted to the proposed models by non-linear least squares methods (quasi-Newton), in *Microsoft Excel* spreadsheet, using *Solver* complement for parametric estimates, and *Solver Aid* macro [6], [7] for confidence intervals and model consistency (Student's *t* and Fisher's *F* tests, respectively, with α = 0.05 in both cases).

In the most complex cases (models with interactions), the fitting process involved always a progressive hypotheses of contrast, as it is usual in any stepwise multiple regression method, to select the interactive mode providing the most statistically consistent interpretation. Parametric confidence intervals, coefficient of multiple determination, residual bias and sensitivity analysis were applied as selection criteria. An efficient way of proceeding is by following the next steps: 1) calculation of the sigmoidal parameters from the individual responses; 2) use of these estimates as provisional fixed values of the model, and assay of different interaction hypotheses, rejecting those that lead to not statistically significant coefficients; 3) refinement of the model by recalculation, now allowing the variation of all the accepted parameters.

Although the initial number of parameters is high, it means only a high number of potential alternatives, some of which are mutually exclusive, and others easily rejected in the course of the fitting to a concrete data set. Trying of initial values is facilitated by follow up of the variations produced by the *Solver* results in graphic displays of response surface and residuals, and convergence is usually immediate. Details about experimental design are provided as *Supporting Information* (see Figure S1).

### 2. Null interaction, synergy and antagonism. IA and CA hypotheses

In any system (as defined in the Bertalanffy's sense: a set of interacting elements), an important and characteristic problem is to know whether the joint effect of two or more elements on the system behaviour is deducible from their individual effects. This issue, with a long history of controversy whose first known attempt goes back to Aristotle, is often stated by replacing «deducible» with «the sum», what leads to define the notions of synergy and antagonism as those interactions by virtue of which the joint effect of two (or more) effectors is greater (synergy) or lesser (antagonism) than the sum of the individual effects.

For examining the meaning of this non-acceptable statement, let us to consider a system in which two effector elements (E_1_ and E_2_) can act, and let us to denote the possible behaviours or responses of such a system as R_1_ and R_2_ (if only E_1_ or E_2_ are present), R_1,2_ (if E_1_ and E_2_ do not interact), and R_1&2_ (if E_1_ and E_2_ interact). Under these conditions, to obtain any initial analysis on the possible interactions, it is an essential requirement to compare the responses R_1,2_ against R_1&2_, defining synergy and antagonism respectively, as those interactions in which R_1,2_<R_1&2_ and R_1,2_>R_1&2_.

It is obvious that the key aspect to assess any interaction is the response of the system in the absence of interactions (the null interaction). Thus, to define the null interaction, any mechanism must be postulated as underlying any specific behaviour of the system. The addition is the simplest mechanism, and its most immediate options suppose that the added magnitudes can be the effectors (acting as one alone), or the effects on the system of their independent actions (the responses of the system to such actions).

Now, if we imagine that the response has a superior limit, as it happens, for example, in the case of the death-survival alternative in a microbial population under increasing doses of two toxics. If the added magnitudes are the concentrations of the toxics, the response obtained, would be identical to the resulting response values of a single toxic dose –although it can be expressed as a function of two independent variables–. The additive response is more problematic, since it is obvious that if one cell dies when one of the doses reaches a given level, it will die independently on the level of the other dose, simply because it cannot die twice.

These two types of sum are the foundations of the two basic accepted modes for describing the joint action of two effectors under null interaction conditions: concentration addition and independent action. Although both can be applied –at least in theory– to any number of effectors, here will be discussed in their simplest forms, for two effectors (their generalizations are, anyway, immediate).

#### 2.1. Independent action hypothesis

It supposes that the effectors act through different mechanisms, whose asymptotic maxima are reached through statistically independent phenomena. Under this premise, the probability theory allows to define the response as the sum of the probabilities of the individual phenomena minus the probability of their joint occurrence [1], [8]. Consequently, if *R*_c_ is the response to the joint action of *c*_1_ and *c*_2_ concentrations, and *R*_c1_ and *R*_c2_ the individual responses at the same concentrations, it can be established:
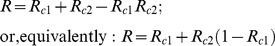
(1)


An expression easily generalizable to more than two effectors is obtained by writing the first *R*_c1_ in the second member of (1) as 1−(1−*R*_c1_):
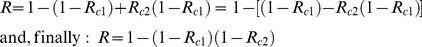
(2)


#### 2.2. Concentration addition hypothesis

In its classical formulation [2], [3], null interaction is not defined as a relation between the individual responses, but through the following criterion: since the concentration (*c*) of an effector whose action obeys the equation *R* = *f*(*c*) can be considered as a fictitious combination of *c*_1_ and *c*_2_ concentrations (*c* = *c*_1_+*c*_2_), it is obvious that the response to *c* will be described by the equation *R* = *f*(*c*), with *c* = *c*_1_+*c*_2_. If the response to a mixed dose of two effectors behaves as the response to the “mixed” dose of the same effector, it is accepted that the interaction between them is null, implying that any effector concentration can be substituted by the equieffective concentration of the other one.

**Figure 1:**
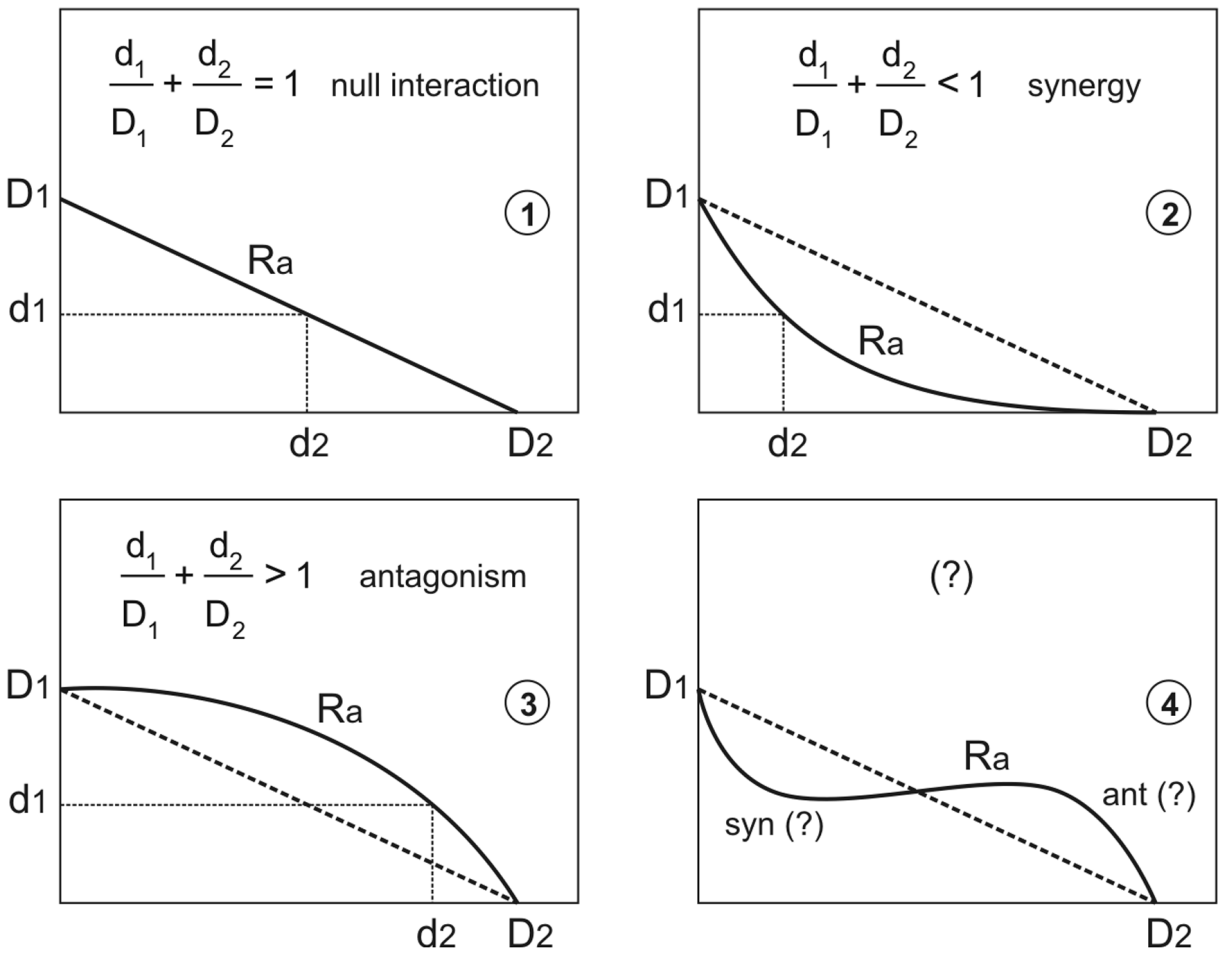
Isobole of a response R_a_. In 1, 2 and 3 the geometric logic underlying the analysis of the CA hypothesis through the equation (4) is shown. Type 4 isoboles arise in many real responses corresponding to the IA hypothesis with null interaction, and illustrate the limitations of the relation between factual and formal aspects of isobole analysis beyond a particular case of the CA mode of action (see results, section 5).

The conventional practice avoids an explicit algebraic formulation when evaluating the joint response of effectors, using the isobolographic analysis [9] (or lines on the plane of the independent variables that represent the dose combinations that produce an equal response) to solve the lack of a clear mathematical process. Thus, in the isobolographic analysis, if *D*_1_ and *D*_2_ are the doses of two effectors that produce the individual response *R*_a_, and *d*_1_ and *d*_2_ any dose combination that produces the same joint response *R*_a_ (Figure 1), under null interaction conditions the isobole of the response *R*_a_ will be necessarily described by the following lineal equation:
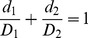
(3)


Consequently, if the individual DR models are *R*_i_ = *f*_i_(*D*_i_) and their reciprocal functions *D*_i_ = *g*_i_(*R*_i_) exist, it can be established that:
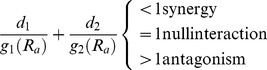
(4)


In other words: straight isoboles indicate null interaction, and concave and convex up isoboles indicate synergy and antagonism, respectively. The dimensionless quotients *d*_i_/*g*_i_(*R*_a_) are called toxic units and represent the relative contribution of each effector to the joint response *R*_a_.

### 3. Some problems associated with the AI-AC approach

A first unsatisfactory aspect of this approach is the difference between the formal criteria applied to each mode of action. IA hypothesis proposes an explicit response surface model, but a general agreement does not exist about the ways in which synergy and antagonism must be formulated. CA hypothesis avoids the explicit model, but equation (4) is accepted as a criterion for detecting synergy and antagonism. This criterion, however, is not transferable to the IA framework, whose mathematical form prevents straight isoboles in null interaction if –as it occurs in most DR relationships– the individual responses *R*_1_ and *R*_2_ are not proportionally constant. As a general rule, IA isoboles in null interaction are convex up at low doses, concave up at high doses, and with two branches of opposite curvature in a transitional zone when *R*_1_≠*R*_2_ (Figure 1). But the factual meaning of this formal property cannot be attributed to synergy or antagonism, only implying that the probability that at least one dose is lethal is low at low doses and high at high doses (another outcome of the statistical independence, as the above mentioned impossibility of dying twice).

In CA hypothesis, as already pointed out, the absence of an explicit mathematical model is another disadvantage. A measure of the sign and degree in which an isobole deviates from linearity is provided by (4), and isoboles with a variable degree of curvature and asymmetry can be defined by using alternative expressions as equation (5) described by [10] or equation (6) described by [11].
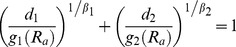
(5)

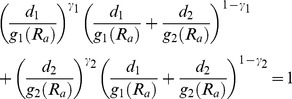
(6)where the additional parameters β or γ are more precise indexes of synergy and antagonism. Although these equations would enable the construction of isobole maps by solving them numerically, the procedure is laborious and in practice is usually applied only to the isobole at the half-maximum response. Furthermore, in this approach a homogeneous isobolic curvature is postulated for the whole considered domain, a property that as we shall see later, is not necessarily true.

Some joint responses to chemically similar/dissimilar effectors were suitably described with CA/IA models, respectively [12], [13]. However, there are evidences, as well, that these modes of action are not always obeyed by the reality. Jonker et. al [14] have proposed in each case, besides synergy and antagonism, other deviations from null interaction which were defined *a priori* as effects depending on the absolute dose levels or dose ratios, thus enabling synergistic and antagonistic displays on different regions of the same response surface. But even so, the reality seems to be richer: in a revision of 158 cases [15] was found that 20% of the responses can be adequately predicted by IA, 10% by CA, another 20% admitted both models and half of the cases were not correctly described with either of them. Moreover, neither of the models was significantly better than the other on assessing synergy and antagonism at the 50% effective doses.

**Figure 2:**
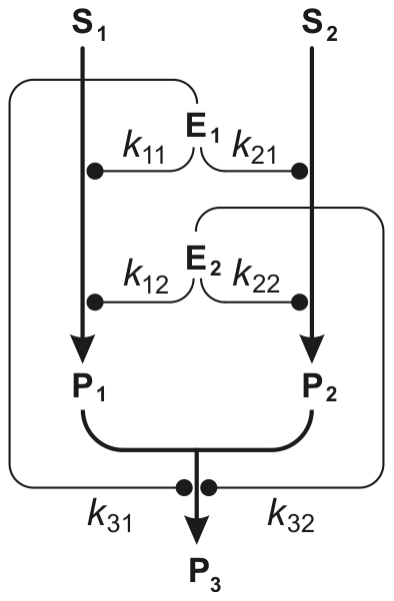
Ambiguity of the IA-CA dualism. Rates of the enzymatic reactions yielding products P_1_ to P_3_ from substrates S_1_ and S_2_ are affected by the inhibiting effectors E_1_ and E_2_ with the specified inhibition constants *k*_ij_. Under these conditions, responses measured as drops of the levels of any of the products are dependent of the nature (competitive, non-competitive, acompetitive) of the inhibition and the values of the inhibition constants. But any result can be unambiguously attributed to IA or CA modes of action.

These results are not really surprising. Figure 2 represents a simple hypothetical metabolic pathway that can lead to a set of different situations depending on the considered inhibition mechanisms (competitive, non-competitive, acompetitive), the kinetic constants that are null, the values of the rest of such constants and the definition of the response (individual or simultaneous drops of the products P_1_, P_2_ or P_3_). But even without including an experimental error, none of these possible situations obeys unambiguously IA or CA modes of action. In our laboratory, these ambiguities have been detected not only in the joint action of hydrocarbons and dispersants on the larval growth of sea urchin [16], but also, as we will see, in the simpler context of the oxidation inhibition of a substrate by the joint action of two antioxidants.

### 4. DR model for a single effector

The natural form of a DR model is a cumulative (mass) probability function, and it translates the response of a population with a given sensitivity distribution to an effector. Four additional conditions seem reasonable as well: 1) the model should have an explicit algebraic form; 2) it should be lacking of an intercept (null response at null dose); 3) an asymptote equal or lesser than 1 should be enabled; and 4) the parameters with important factual meaning should be explicitly included, to facilitate the trial of initial values and the calculation of confidence intervals when non-linear fitting methods are applied.

Although normal and log-normal distributions have been the basis of the classical DR analysis, they have the disadvantage of lacking of an explicit form for their mass functions. Logistic-type equations are more useful and they can be expressed in forms easily modifiable to comply with the above mentioned conditions [17], [18]. However, their derivatives (their density functions) show only right bias, which can be a restriction scarcely realistic. Another option is the Gompertz [19] equation, but its use is prolix, especially with the modifications that are required to apply in the DR context. The mass function of the Weibull distribution [20] can be expressed in a suitable reparametrized form [21]–[23], providing the adequate DR analytical model, whose expression can be write as: 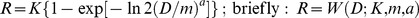
(7)where *D* is the dose, *R* the response (with *K* as asymptotic maximum, not necessarily 1), *m* the dose producing half-maximum response, and *a* a shape parameter related to the maximum slope of the response (*r*_m_) by:
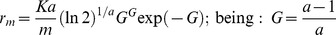
(8)


It should be noted that *m* is the abscissa of the inflection point, which represents the accumulated modal response. The basic parameter of the DR analysis is ED_50_ or EC_50_, defined as the effective dose for the 50% of the assay population. Thus, *m* and EC_50_ are coincident values if the maximum response implies the whole population (*K* = 1). The reciprocal function of equation (7), which is a requirement when performing the isobole analysis in the CA hypothesis, it can be written as:
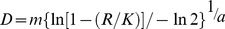
(9)


The use of the equation (7) as DR model is interesting for several reasons. Its density function (the sensitivity distribution of the population) can be symmetrical or asymmetrical with right or left bias, which makes it very versatile. It produced the best fittings, among the above mentioned alternatives, when it was applied to the simulations that will be described in the next sections, and this result was repeatedly confirmed before by experimental data [21], [24]–[27]. Moreover, the Weibull distribution is the conventional model for the failure of complex devices, making the equation more attractive because it unifies phenomena in which underlies a profound analogy.

### 5. Simulation of the response to a single effector

Since the basic sigmoidal profile of the DR relationships translates a macroscopic, statistical phenomenon, it should be possible to simulate it as a result of the microscopic behaviour of a population of elementary biological entities, which we will call cells. Such a simulation can be carried out on the following basis:

B1.One cell is defined by means of three aleatory magnitudes: ρ, or number of receptors of an effector, α, or number of active receptors –ready for linking the effector– in a given instant, and λ, threshold or minimum number of active receptors that must be linked to an effector to produce a response.

B2.The dose *D* is defined as the number of effector units per cell, accepting that every unit is capable of linking to one receptor.

B3.The cell response *r* is limited to two modalities: death (*r* = 0) and survival (*r* = 1), obeying the following logical rule:



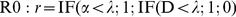
(10)or, as a Boolean proposition (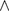: and, 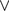: or, 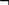: no, 1: true, 0: false):
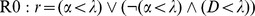



Notice that α<λ implies *r* = 1 at any dose. It translates possible limitations in the effector bioavailability, resistant cells or other conditions which, relatively frequent in DR assays, produce lesser than 1 asymptotes.

Although ρ (highest limit of α) is interesting in some cases, hereafter it will be omitted without loss of generality, and a cell will be defined through the pair α, λ. Thus, if we assign to α and λ probability distributions defined by their mean and variance values, we can create, in a spreadsheet, a virtual cell population whose sensitivity distribution depends on the parameters α and λ. The population response R to an increasing dose series is simulated by applying the rule (10) to each cell and defining R = 1−(S/Y) when S cells, of a total number Y, survive at a given dose. The typical shape of the response arises even at moderate population sizes (Y∼100) and becomes highly stable when Y≥1,000. At low population sizes, the variability of the result represents a simulation of the natural variability, the experimental error, or both.

These premises define the minimum complexity of a system able to generate a sigmoidal response and, despite their schematic character, they produce a great variety of profiles, depending on the α and λ parameters. Although the microscopic solution of the system is determined by such parameters, the obtained profiles can be macroscopically described by the conventional equations of the DR analysis.

The use of the Weibull distribution for defining α and λ is not essential. With normal, log-normal, Poisson, binomial, or even uniform distributions, the best descriptions of the responses are obtained, as it has been mentioned. However, distributions with domain [0,∞) are obviously preferable, since low means and high variances in (−∞,∞) distributions lead to a finite probability of negative values of α and λ, which lacks of physical meaning.

### 6. Simulation of perturbations of the response to an effector

**Figure 3:**
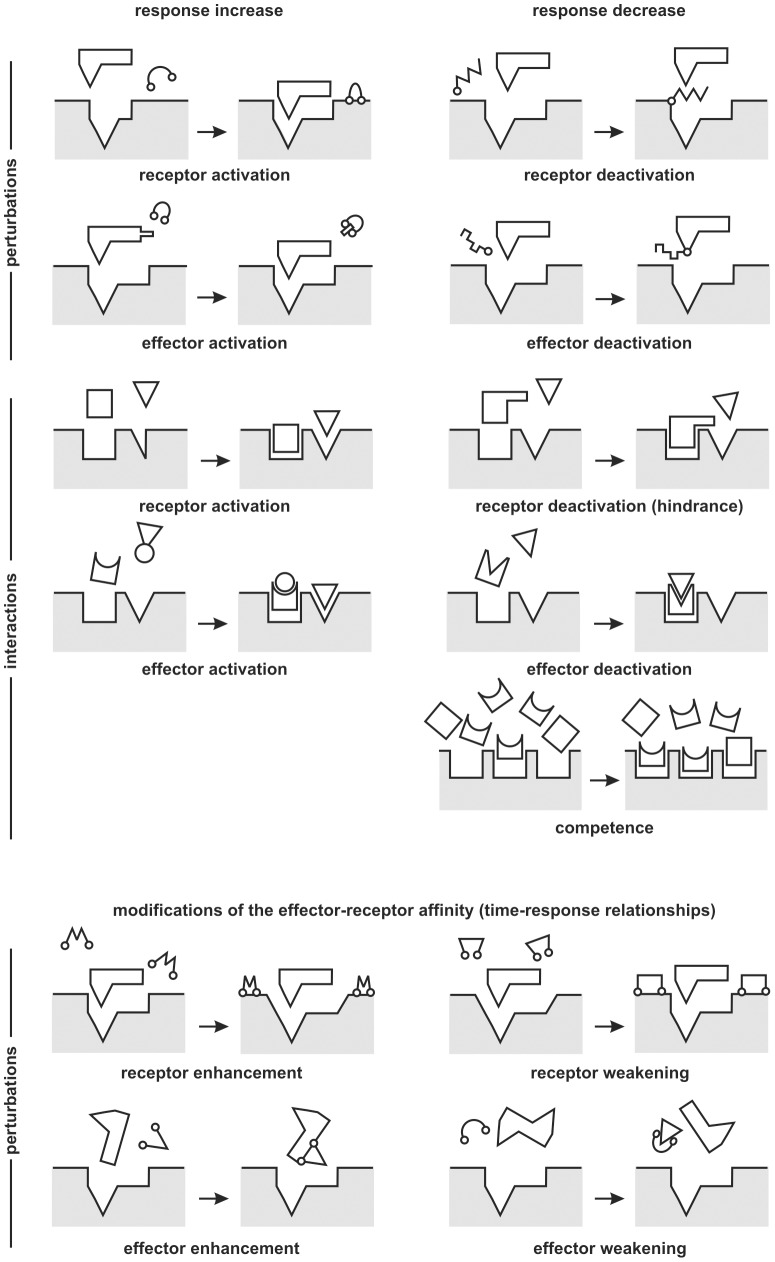
Some key-lock analogies. Possible response modifications involving alterations of the effective dose or the number of active receptors are illustrated (see Table 1). Notice that the alterations of the effector-receptor affinity (for simplicity reasons only perturbations are illustrated) do not modify the response to a given dose, but the response to a given time.

Using the elements above defined, we will admit that a perturbator does not produce a response by itself, but it can modify the response to an effector by altering the number of active receptors (α), the threshold (λ) or the effective dose corresponding to the nominal dose (*D*) (hereafter α, λ and D-perturbations, respectively). α and D-perturbations can be illustrated in molecular terms through the well-known key-lock analogies (Figure 3). λ-perturbations require to suppose an intermediate fast process modifying the cell sensitivity.

All of them can be exemplified by common physiological mechanisms as those that take place in trans-membrane proteins, second messengers or enzymatic systems, in which any DR assay allows to distinguish at least between α and D-perturbations. Indeed, in the presence of an excess of effector, a moderate increment of a perturbator modifies or not the response depending on whether the perturbation acts over the receptors or the effector.

The effect of these perturbations on the response can be simulated by using the rule R0 (10) and adding a vector that represents increasing perturbator concentrations, as well as a criterion for modifying the values of α, λ or *D* as a function of the perturbator concentration. Since direct or inverse ratios are the simplest criteria, a perturbation term can be formulated as:
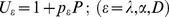
(11)where *P* is the perturbator concentration, *p*_ε_ the proportionality coefficient, and the ε subscript indicates the element affected by the perturbation. The term *U*_ε_ multiplies or divides the values of *D*, α or λ depending on the effect that we are trying to achieve, and it can be replaced by any other algebraic expression able to describe any other type of alteration.

Now, it can be pointed out that if a DR curve is sigmoidal because the most sensitive elements of a population die at lower doses than the most resistant ones, therefore a time-response curve will be sigmoidal if the sensitivity distribution of the population is translated into responses at shorter or longer times. Although this problem will not be considered here, the time-course of the response could be treated also in terms of molecular interactions, which in this case, would accelerate or delay the progress of the effect (Figure 3). In some fields –*i.e*. marine toxins–, the time of death of a target organism often replaces the response in the framework of the empirical dose-time death models [28]–[31]. We do not allude here to this approach, but to the time-response relationships, which can be described by using the same models as DR analysis [26].

### 7. Simulation of the response to two effectors

In this case, the perturbator must be replaced by a second effector, but the essential issue is the need to include in the simulation algorithm, even in the absence of any interaction, any rule about the joint action. The simplest hypothesis in this regard leads to admit that: 1) the receptors are specific of the effectors; 2) the cell response is *r* = 0 if any of the doses exceeds the corresponding threshold and there is a sufficient number of receptors. Thus, the rule is:
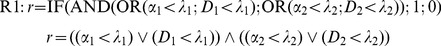
(12)


These conditions are reminiscent of IA hypothesis and in fact, as we shall see later, they produce the typical IA response surfaces. Now then, both conditions can be denied, what seems quite reasonable for the second one [32], since it prevents to accept that two biological subsystems –*e.g*. glycolysis and β-oxidation– can be affected at individually sub-lethal, but jointly lethal levels. By denoting the first condition (specificity) as **S^+^** and the second one (independence) as **I^+^**, three additional rules, besides **S^+^I^+^**, can be considered:

**S^+^I^−^**. It admits that 1) the effect Gi of a dose Di below threshold λi is Gi = Di/λi; 2) Gi values are additive; 3) r = 0 if G1+G2≥1 and there enough receptors:
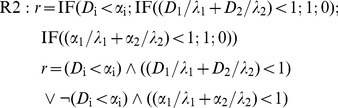
(13)


**S^−^I^+^**. It admits that any effector has access to the whole of the receptors (α_1_+α_2_), what produces competence if they are insufficient. Competence depends on factors as the relative doses of the effectors, their diffusivity or their affinity for the receptors, but to simplify, only relative doses will be considered here: *C*_1_ = *D*_1_/(*D*_1_+*D*_2_) and *C*_2_ = *D*_2_/(*D*_1_+*D*_2_). Thus, the number of receptors linked to *D*_i_ will be *C*_i_(α_1_+α_2_), what leads to the rule:
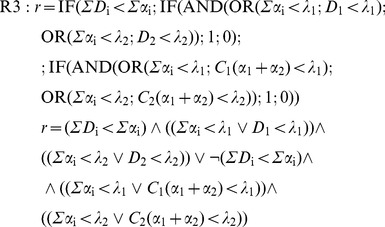
(14)


**S^−^I^−^**. It admits simultaneously competence (if the receptors are insufficient) and additivity of below-threshold effects. The rule is:
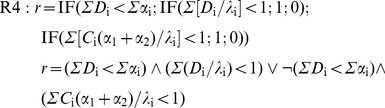
(15)


None of these rules implies the sum of the doses required by the CA hypothesis. This condition plays down the receptor specificity, but again the competence arises if the receptors are insufficient. If both effectors have the same threshold, the response is not modified by the competence. If the thresholds are different, the number of occupied receptors will depend on the relative doses and will be *C*_i_(α_1_+α_2_), turning the dose addition effect in below-threshold addition effect: *G*_i_ = *C*_i_(α_1_+α_2_)/λ_i_. Therefore, two rules are possible, any of which produces the response surfaces with straight isoboles that characterize the null interaction in the CA hypothesis:

R5a: Without competence (equal thresholds):
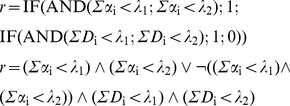
(16)


R5b: With competence (different thresholds):
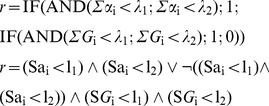
(17)


### 8. Inherent and accidental mechanisms

Concrete assumptions (about specificities, thresholds, competence and dose or effect addition) as those included in the rules R1 to R5 are indispensable for any joint action hypothesis, and they represent mechanisms that are inherent to a given null interaction model. Over these minimal inherent modes of action, accidental mechanisms (interactions) can be superimposed, in which each effector can perturb the response to each other by modifying the number of active receptors, the threshold or the effective dose corresponding to the nominal dose. Thus, when the effector E_1_ perturbs the response to E_2_, we can write an interaction term like (11):
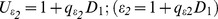
(18)which is included into the rules R1 to R5, as a factor or divisor of *D*_2_, α_2_ or λ_2_.

The interactions can be unidirectional (E_1_ alters some factor related with E_2_, but E_2_ does not alter E_1_ accordingly) or reciprocal (mutual alterations), and these last ones can or cannot be symmetrical (the same or different strength in both directions). Whereas the reference of a perturbation is the response in the absence of perturbator, the reference of an interaction is the inherent mechanism or null interaction. This implies that a given interaction has different consequences depending on the inherent mechanism and the frame of application. A systematics in this regard is proposed in Table 1, which attempts to preserve the main senses of the usual terminology [33], and whose categories allow simulations and specific formal descriptions. Although this terminology can be used without ambiguity, it seems preferable to simplify it by defining stimulation/inhibition as perturbations that increase/decrease the response to an effector, and sinergy/antagonism as interactions that increase/decrease the joint response to two effectors with respect to the response promoted by the null interaction.

**Table 1:** A possible systematics on the modifications of the response to an effector.

object of the modification	value	modifier do not promotes response (perturbations)	modifier is another effector (interactions)
effective dose corresponding to the nominal dose	higher	POTENTIATION	SYNERGY
	lower	DEPRESSION	ANTAGONISM
number of active receptors	higher	STIMULATION	ACTIVATION
	lower	INHIBITION	INACTIVATION
threshold	higher	INSENSITIZATION	ATTENUATION
	**lower**	**SENSITIZATION**	**ENHANCEMENT**

When the modifier is a second effector (interactions), unidirectional and reciprocal effects can be considered in every case. For details, see text.

## Results

### 1. Perturbations of the response to a single effector

**Figure 4:**
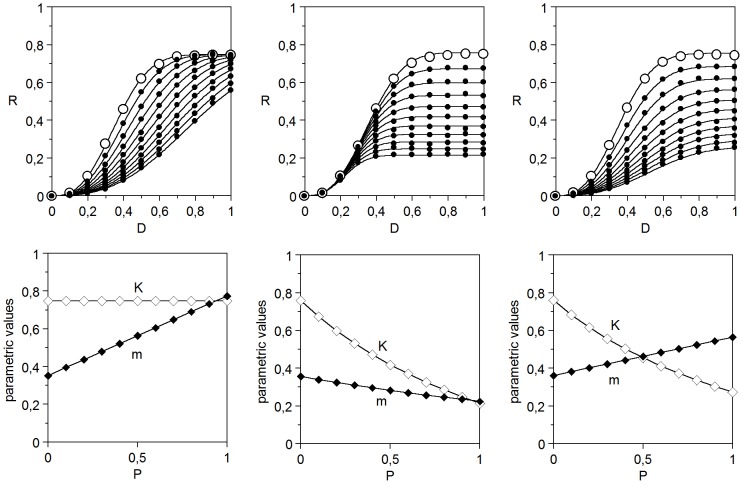
Effect of a perturbator on the response (*R*) to a same dose series (*D*) of an effector and the parameters of the model (22). The three cases in which the perturbation depresses the response are illustrated: reduction of the effective dose corresponding to the nominal one (left), reduction of the number of active receptors (canter), and increase of the threshold (right). Dots are simulated results in the absence (○) and presence (•) of increasing concentrations of the perturbator, and lines the respective fittings to model (22). See also tables 2 and 3.

In Figure 4 the three conditions that depress the response simulations to an effector in the presence of increasing concentrations (*P*) of a perturbator are shown (decrease of the effective dose, decrease of the number of active receptors and increase of the threshold). The individual fittings to the resulting profiles with the model (7) proved that, in each series, the estimates of *K* and *m* parameters varied as a function of *P* in specific ways (Table 2), thus enabling to identify the underlying perturbation. Although the maximum slope varied as a consequence of the variations of *K* and *m*, in agreement with (8), the parameter *a* remained constant in all cases.

**Table 2:** Variations (+: increase; −: decrease; 0: no change) in the parameters of the response to an effector, as described by the equation (7), due to the presence of an agent which produces the specified perturbations.

	alteration due to the perturbator					
	effective dose		active receptors		threshold	
	higher	lower	higher	lower	higher	lower
K	0	0	+	−	−	+
m	−	+	+	−	+	−

Maximum slope, *r_m_*, varies according to (8), but the parameter *a* remains constant in all cases.

Such a constancy of *a* is not surprising, since the simulations with the rule R0 prove that the variation of this parameter is related with the variations of the variances of α and λ, a condition that was not considered in any case. Such a restriction simplifies the analysis and is not arbitrary. From the microscopical point of view adopted for the effector and perturbator actions, variations in the number of receptors and threshold are clear possibilities, but it is more difficult to justify an action on a populational property such as the variance.

#### 1.1. The perturbation function

**Figure 5:**
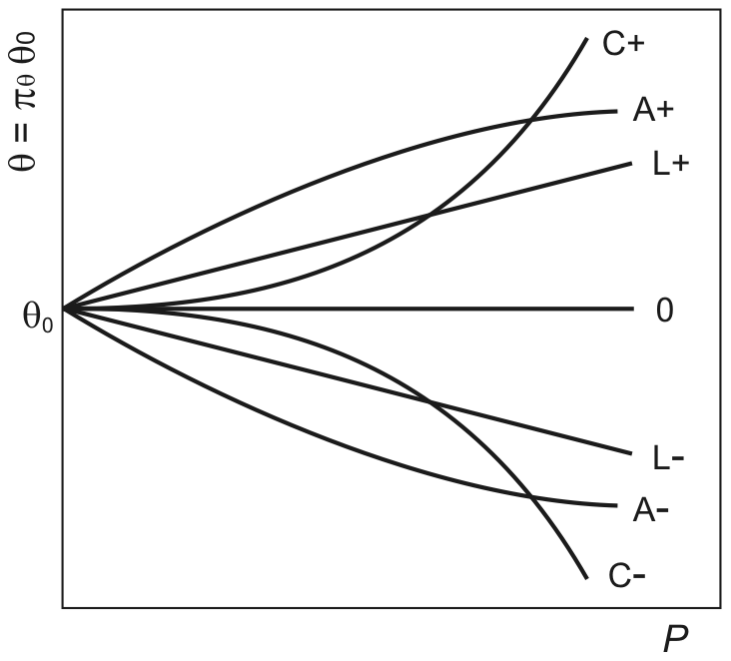
Possible variations of a parameter (θ) of the response to an effector, as a function of the concentration of a perturbator (*P*). Any of the functions (19), (20) and (21) can produce all the profiles. Parametric value can be increased (+) or decreased (−), with constant (L), decreasing (A) or increasing (C) slope.

To obtain a simultaneous solution for every series of profiles, an auxiliary function (a perturbation function π_θ_) is required for describing the possible variations of any parameter θ as a function of *P* (Figure 5). This can be achieved by means of different biparametric (exponential, polynomial or hyperbolic) expressions:
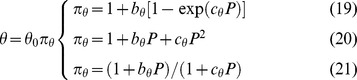
where the θ subscript represents the modified parameter (*K*, *m*), θ_0_ is the parametric value when *P* = 0, and the pairs *b*_θ_, *c*_θ_ are fitting coefficients. It should be noted that, in the absence of perturbation, the first function requires *b*_θ_ = 0 and *c*_θ_ = 1, whereas the other two require *b*_θ_ = 0 and *c*_θ_ = 0. Moreover, the condition of the third function a singularity is obtained if *c*_θ_ *P* = −1. In order to avoid it, when *P* is coded in the interval [0,1] it is advisable to include the restriction *c*_θ_>−0.999 in the fitting algorithm. Thus, the model (7) turns into:
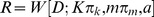
(22)


With any of the mentioned forms of π_θ_, the (22) led to excellent simultaneous fittings, which confirmed the specificity of the parametric variations found in the individual descriptions. Figure 4 and Table 3 show the results obtained with the hyperbolic function (21), which will be the form used now on. The description of the inhibition of the haemolytic activity of palytoxin by ouabain [22], illustrates the application of equation (22) for perturbations involving a time-response profile.

**Table 3:** Simulation conditions of the responses to an effector as perturbed according to the three modalities that cause response drop, and respective fittings to the model (22).

α (mean; sd)	120;48	120;48	120;48
λ (mean; sd)	80∶32	80∶32	80∶32
p_ε↑↓_	p_D↓_ = 0.006	p_α↓_ = 0.006	p_λ↑_ = 0.006
K	.0.748±0.002	0.756±0.004	0.760±0.005
m	0.352±0.001	0.353±0.002	0.361±0.003
a	2.696±0.015	2.685±0.042	2.685±0.045
b_K_	-	−0.542±0.013	−0.400±0.028
c_K_	-	0.631±0.038	0.671±0.061
b_m_	1.198±0.009	−0.385±0.013	0.564±0.030
c_m_	-	-	-
adj. r^2^	0.9999	0.9998	0.9994

r^2^: correlation coefficient between observed and predicted results. See also Table 1 and Figure 4. Number of active receptors (α) and threshold (λ) are defined by means of aleatory Weibull numbers. Doses and perturbator concentrations varie in the natural domain [0-(20)-200] and are coded in the domain [0-(0.1)-1]. p_ε_ coefficients –which operate on natural values of D, α and λ– are those defined in (11). Arrows indicate increase (↑) and decrease (↓) of the affected element.

When the model (22) is satisfied with uniparametric π_θ_ (*b*_θ_ = 0 or *c*_θ_ = 0), the relationships between confidence intervals (ci) and parametric values are approximately of the same numerical size in all the cases. When *b*_θ_≠0 and *c*_θ_≠0 are required, both ci are penalized by the linear correlation between both coefficients, and therefore, with high experimental error and low number of observations, some *b*_θ_, *c*_θ_ pair can become not statistically significant, even in highly predictive models. This problem is solved if the model is recalculated fixing the value of one of the coefficients and excluding it of the Student test, or –with a small loss of predictive capability– making zero the coefficient of the pair whose suppression does not alter the increasing or decreasing trend of the parametric variation due to the perturbation function.

Finally, in D-perturbations is interesting to observe that two equivalent solutions are possible. One of them is that provided by the model (22), describing the variation of *m* (the only affected parameter) due to the presence of the perturbator. The other one describes directly the variation of the effective dose through the equation:
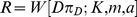
(23)


Later on we will see that this dual solution is not possible in interactions between two effectors.

### 2. The response surfaces to two effectors

Among the five basic types of response produced by the rules R1 to R5, only two of them were in accordance with those corresponding to IA and CA hypotheses, and the diversity increases as interactions are included. Two possible reasons to avoid describing this diversity as unrealistic are because it involves options that are implicit in the same rules that produce IA and CA responses and it translates common physiological mechanisms. In fact, the situation seems to be just the opposite one. As Rovati et. al [34] pointed out regarding the cases of partial agonism, or effectors able to interact with receptors promoting opposite effects, there are responses «that were often disregarded by the experimentalists, or considered as artefacts, in the absence of a biological and/or mathematical theory to justify them».

The parametric variations due to interactions showed the same specific increasing and decreasing trends as those due to perturbations (Table 2), in linear or asymptotic forms (Figure 5) depending on quantitative factors, and such fact is hardly surprising.

### 3. Modelling of interactions under the independent action hypothesis

**Figure 6:**
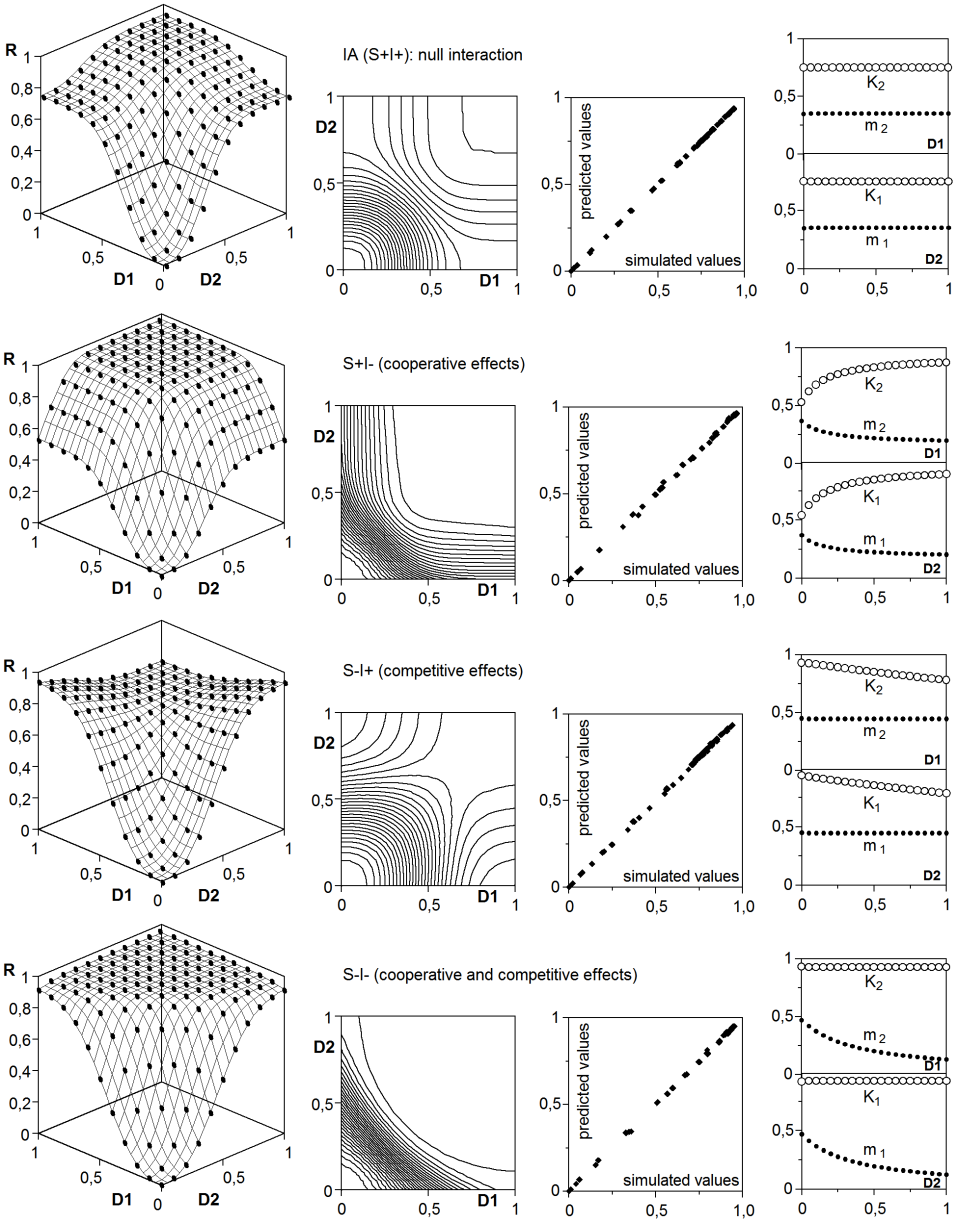
Joint response to two effectors in the four suppositions resulting from combining the two implicit key conditions of the IA hypothesis (see text). In the first column, dots are the result of simulations and surfaces the respective fittings to the model (26). Isobolograms, correlations between observations and predictions and parametric variations (*K*_i_: ○, *m*_i_: •) of the response to an effector as a function of the dose of the another are added. D_1_ and D_2_: doses; R: response. Numerical data in Table 4.

As expected, the response surfaces produced by the rule R1 were consistent with the IA hypothesis (Figure 6, Table 4) and could be accurately described by transferring model (7) to equation (2):
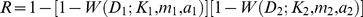
(24)


**Table 4:** Simulation conditions and respective fittings (α = 0.05) to the generalized IA model in the specified examples.

		S^+^I^+^ (independent action)						
		null interaction	antagonism	α-antagonism	λ-antagonism	S^+^I^−^	S^−^I^+^	S^−^I^−^
receptors (α_1_ = α_2_)		120;48	120;48	120;48	120;48	100;40	100;40	95;38
λ_1_ threshold		90;36	80;32	80;32	80;32	95;38	95;38	100;40
λ_2_ threshold		75;30	80;32	80;32	80;32	95;38	95;38	100;40
q [D_1_↑↓D_2_]		-	0.012 [D_1_↓D_2_]	-	-	-	-	-
q [D_2_↑↓D_1_]		-	-	-	-	-	-	-
q [D_1_↑↓α_2_]		-	-	0.01 [D_1_↓α_2_]	-	-	-	-
q [D_2_↑↓α_1_]								
q [D_1_↑↓λ_2_]		-	-	-	0.01 [D_1_↑λ_2_]	-	-	-
q [D_2_↑↓λ_1_]		-	-	-	-	-	-	-
Basic (sigmoidal) parameters	K_1_	0.685±0.002	0.749±0.001	0.750±0.003	0.753±0.002	0.533±0.007	0.935±0.006	0.926±0.005
of the joint response	m_1_	0.382±0.001	0.352±0.001	0.356±0.002	0.355±0.002	0.368±0.006	0.446±0.003	0.473±0.003
	a_1_	2.680±0.027	2.691±0.022	2.661±0.034	2.697±0.034	2.729±0.106	2.667±0.051	2.584±0.047
	K_2_	0.780±0.001	0.747±0.002	0.753±0.004	0.752±0.004	0.535±0.007	0.937±0.006	0.925±0.005
	m_2_	0.337±0.001	0.351±0.002	0.353±0.002	0.355±0.003	0.369±0.006	0.446±0.003	0.471±0.003
	a_2_	2.702±0.022	2.706±0.027	2.657±0.049	2.669±0.053	2.703±0.105	2.658±0.051	2.607±0.048
Joint probability factor	s	1	*1*	*1*	1	1.029±0.005	1.339±0.004	1.054±0.001
Perturbations due to D_1_	b_k2_	0	0	−0.712±0.042	−0.632±0.067	0	0	0
modifying the parameters	c_k2_	0	0	0.936±0.085	1.066±0.120	5.971±0.219	0.198±0.025	0
of the response to D_2_	b_m2_	0	2.434±0.031	−0.574±0.033	1.074±0.072	0	0	0
	c_m2_	0	0	0	0	5.690±0.265	0	2.684±0.111
Perturbations due to D_2_	b_k1_	0	0	0	0	0	0	0
modifying the parameters	c_k1_	0	0	0	0	5.573±0.216	0.193±0.025	0
of the response to D_1_	b_m1_	0	0	0	0	0	0	0
	c_m1_	0	0	0	0	6.097±0.274	0	2.792±0.112
	r^2^	0.9999	0.9998	0.9996	0.9996	0.9994	0.9994	0.9997

**Figure 7:**
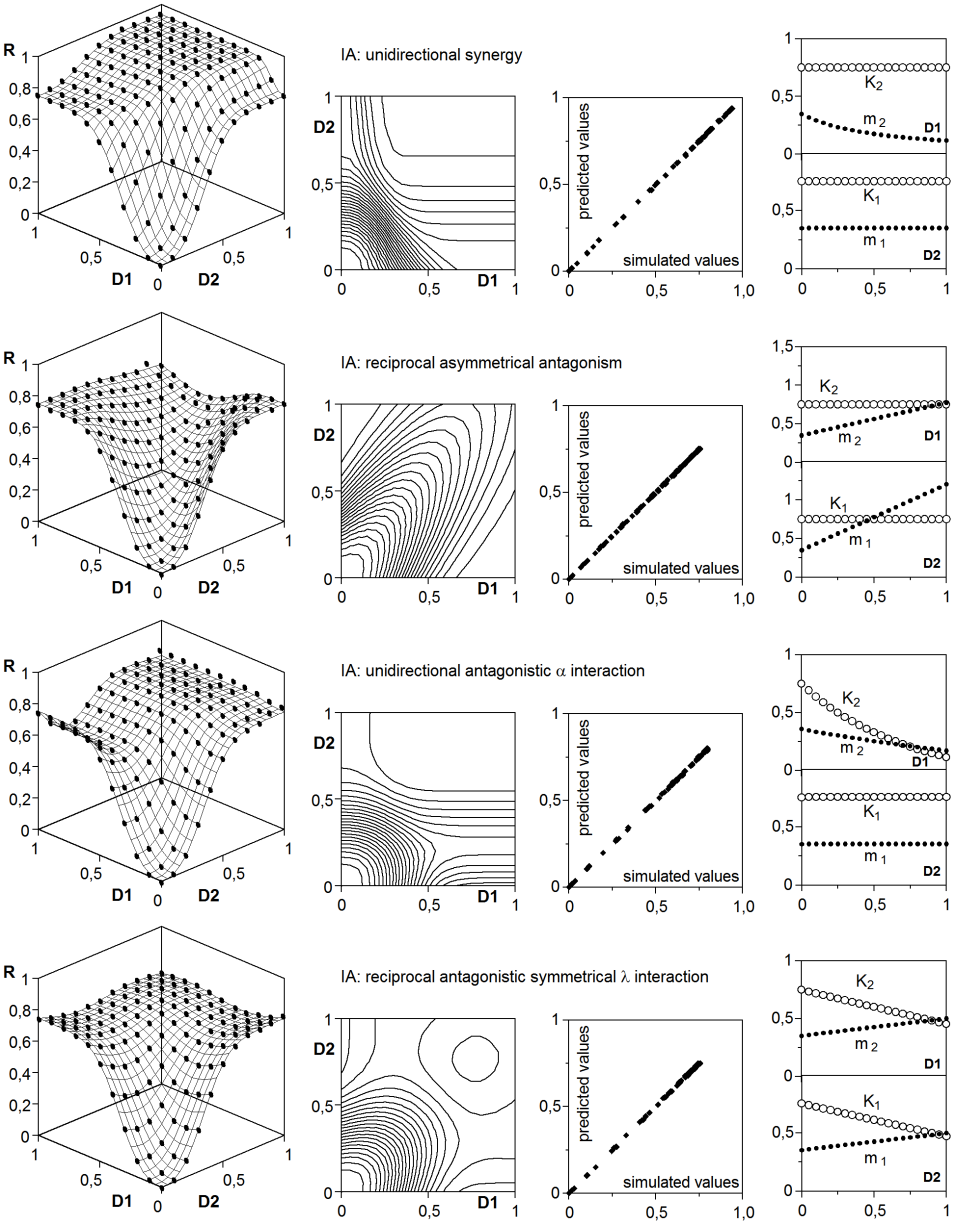
Some examples of joint responses to two effectors under IA mode of action. Concrete types of interactions are specified and adjusted to the generalized model (26). Keys and graphic criteria as in Figure 6. See also Table 4.

**Figure 8:**
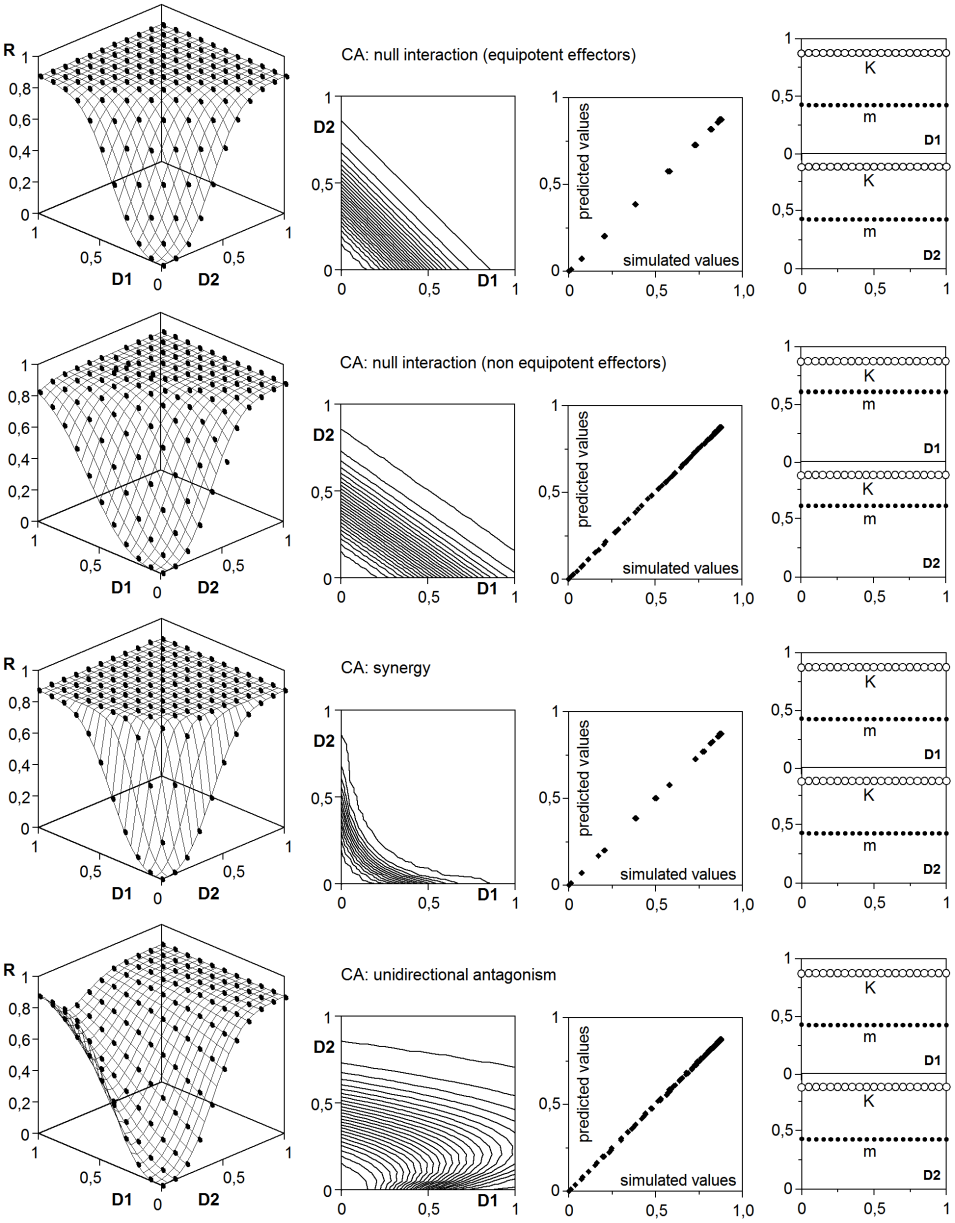
Joint response to two effectors under CA mode of action. Concrete types of interactions are specified and adjusted to the generalized model (32). Keys and graphic criteria as in Figure 6. See also Table 5.

Active receptors (α_i_) and thresholds (λ_i_) were defined as in Table 3. Doses vary within the natural domain [0-(10)-200] and are coded for fittings within the domain [0-(0.1)-1]. *q* coefficients defined by (18): a notation as D_1_↓α_2_ means that the effector E_1_ reduces the value α_2_. r^2^: correlation coefficient between observed and predicted results. See also Figure 6, Figure 7 and Figure 8. Simulation conditions were defined in such a way that produced a typical surface in each case. In S^+^I^+^ (IA) the three basic types of antagonistic unidirectional interactions are shown. Parametric structures of synergistic and reciprocal interactions (some examples in Figure 7 and Figure 8) are immediate by symmetry considerations.

Since each effector can act as perturbator of the response to the other, by altering effective doses, active receptors or thresholds, auxiliary functions π_θi_ can be defined in terms as those applied to the perturbations. Thus, the (24) will be written, in its most complex form as: 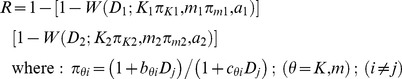
(25)


When this equation was used to describe the corresponding simulations, the specific variations in the parameters *K*_i_ and *m*_i_ led to discriminate all the modalities of interaction (Table 2), whose main types are summarized in Figure 6, Figure 7 and Table 4. For those interactions affecting the effective dose, the dual solution that is possible in the homologous case of the perturbations, here, it would be reduced to parametric variations affecting only to *m*_i_.

#### 3.1. Partial forms of independent action

The R2–4 rules exhibit the three alternatives to the two conditions of R1, which represents the typical independent action. The corresponding simulations showed that unspecific receptors produce a competence that depresses the response, while additive below-threshold effects promote a cooperative effect with an opposite enhancing result. Since the equation (1), that describes IA mode, contains a term (the product of the individual responses) translating the joint probability, it can be supposed that the cooperative and competitive effects could be described by including in (1) a coefficient *s* modifying the contribution of that term.

However, the fitting tests proved that the coefficient *s* is necessary, but not enough, and that accurate descriptions (Figure 6) require parametric structures including interaction terms (Table 4). This seems contradictory with the definitions of inherent mechanism and accidental interaction proposed in the “Simulation of the response to two effectors” section, since S^+^I^−^, S^−^I^+^ and S^−^I^−^ modes (like IA = S^+^I^+^ one) represent inherent mechanisms, without modifications of effective doses, receptors or thresholds. Nevertheless, the lack of specificity in the receptors and the additivity of the below-threshold effects involve that the action of an effector is not indifferent to the presence of the other, what constitutes an interaction, although of a passive character.

Thus, a generalized IA model in its most complex form –in practice several π_θi_ = 1 are expectable–, can be write as:
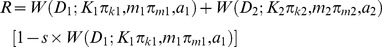
(26)


It should be noted that the need of *s*≠1 detects the relaxation of some of the conditions defining IA mode, but in such a case the identification of possible D, α or λ-interactions become confuse.

### 4. Modelling of interactions under the concentration addition hypothesis

As it has been already mentioned, the application of the CA hypothesis is usually carried out through the isobole analysis. However, the definition of null interaction according to Berenbaum provides the key for establishing an explicit model. Indeed, if the response to a mixed dose of two effectors should behave as a fictitious mixed dose of a single effector, the model is necessarily:
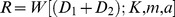
(27)


In fact, the simulations obtained with any of the rules R5 were accurately described by this equation, which produced straight isoboles with equal intersection points on the doses axis (Figure 8 and Table 5). Although the competence affects the parametric values, it does not alter the functional form, making equivalent the two alternatives of the rule R5.

**Table 5:** Simulation conditions and respective fittings (α = 0.05) to the generalized CA model in the specified examples.

receptors (α_1_ = α_2_)		80;32	80;32	80;32	80;32	80;32	80;32	60;24
λ_1_ threshold		120;48	120;48	120;48	120;48	120;48	120;48	120;48
λ_2_ threshold		120;48	120;48	120;48	120;48	120;48	120;48	120;48
tox (D_2_)		1	0.7	1	1	1	1	1
q [D_1_↑↓D_2_]		-	-	0.02 [D_1_↑D_2_]	0.04 [D_1_↓D_2_]	0.02 [D_1_↓D_2_]	-	-
q [D_2_↑↓D_1_]		-	-	0.02 [D_2_↑D_1_]	-	0.01 [D_2_↓D_1_]	-	-
q [D_1_↑↓α_2_]		-	-	-	-	-	0.004 [D_1_↓α_2_]	-
q [D_2_↑↓α_1_]		-	-	-	-	-	0.001 [D_2_↓α_1_]	-
q [D_1_↑↓λ_2_]		-	-	-	-	-	-	0.02 [D_1_↓λ_2_]
q [D_2_↑↓λ_1_]		-	-	-	-	-	-	-
		null equipotent	null non-equipotent		unidirectional	recipr. asymm.	recipr. receptor	recipr. threshold
		interaction	interaction	synergy	antagonism	antagonism	modification	modification
Basic parameters of the	K	0.874±0.0006	0.874±0.0006	0.873±0.0004	0.874±0.001	0.874±0.002	0.915±0.007^a^	0.700±0.006^a^
joint response	m	0.426±0.0007	0.610±0.0013	0.426±0.0009	0.427±0.001	0.426±0.001	0.422±0.002^a^	0.390±0.003^a^
	a	2.772±0.0160	2.786±0.0138	2.776±0.0181	2.784±0.016	2.778±0.016	2.850±0.040^a^	2.813±0.055^a^
perturbations due to D_1_	b_2_	0	0	7.955±0.075	0	0	0	0
modifying the actual dose D_2_	c_2_	0	0	0	7.986±0.068	4.009±0.024	0	0
perturbations due to D_2_	b_1_	0	0	0	0	0	0	0
modifying the actual dose D_1_	c_1_	0	0	0	0	2.006±0.012	0	0
Relative potency factor	u	1	1.428±0.005	1	1	1	1	1
Perturbations due to D_1_	b_k2_	0	0	0	0	0	0	4.325±0.297
modifying the common parameters	c_k2_	0	0	0	0	0	0.265±0.008	2.738±0.192
	b_m2_	0	0	0	0	0	0	−0.611±0.030
	b_m2_	0	0	0	0	0	0	0
Perturbations due to D_2_	b_k1_	0	0	0	0	0	0	0
modifying the common parameters	c_k1_	0	0	0	0	0	0.116±0.007	0
	b_m1_	0	0	0	0	0	0	0
	b_m1_	0	0	0	0	0	0	0
	r^2^	0.9999	0.9999	0.9999	0.9998	0.9998	0.9992	0.9994

**Figure 9:**
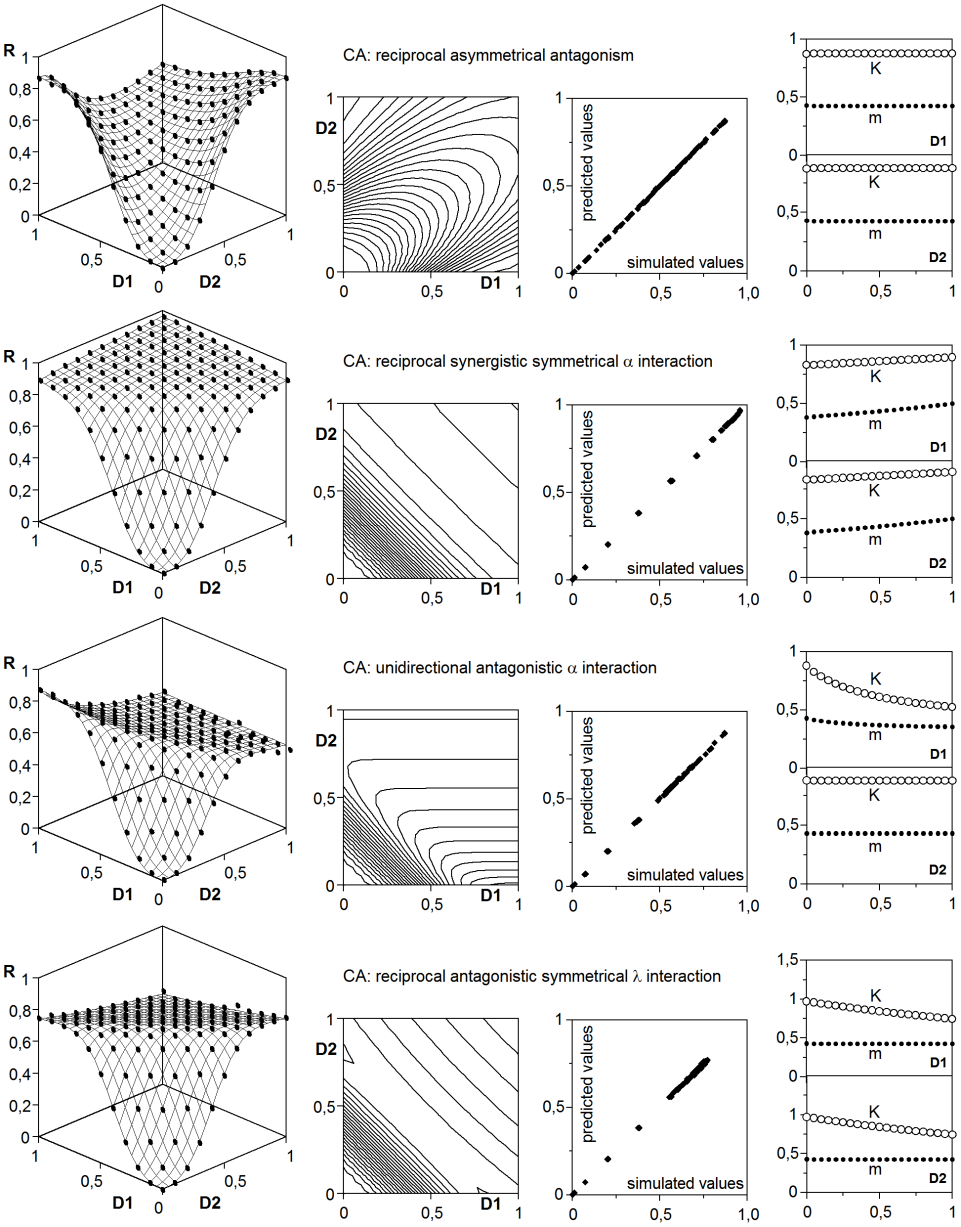
More examples of joint responses to two effectors under CA mode of action. Concrete types of interactions are specified and adjusted to the generalized model (32). Keys and graphic criteria as in Figure 6. See also Table 5.

**Figure 10:**
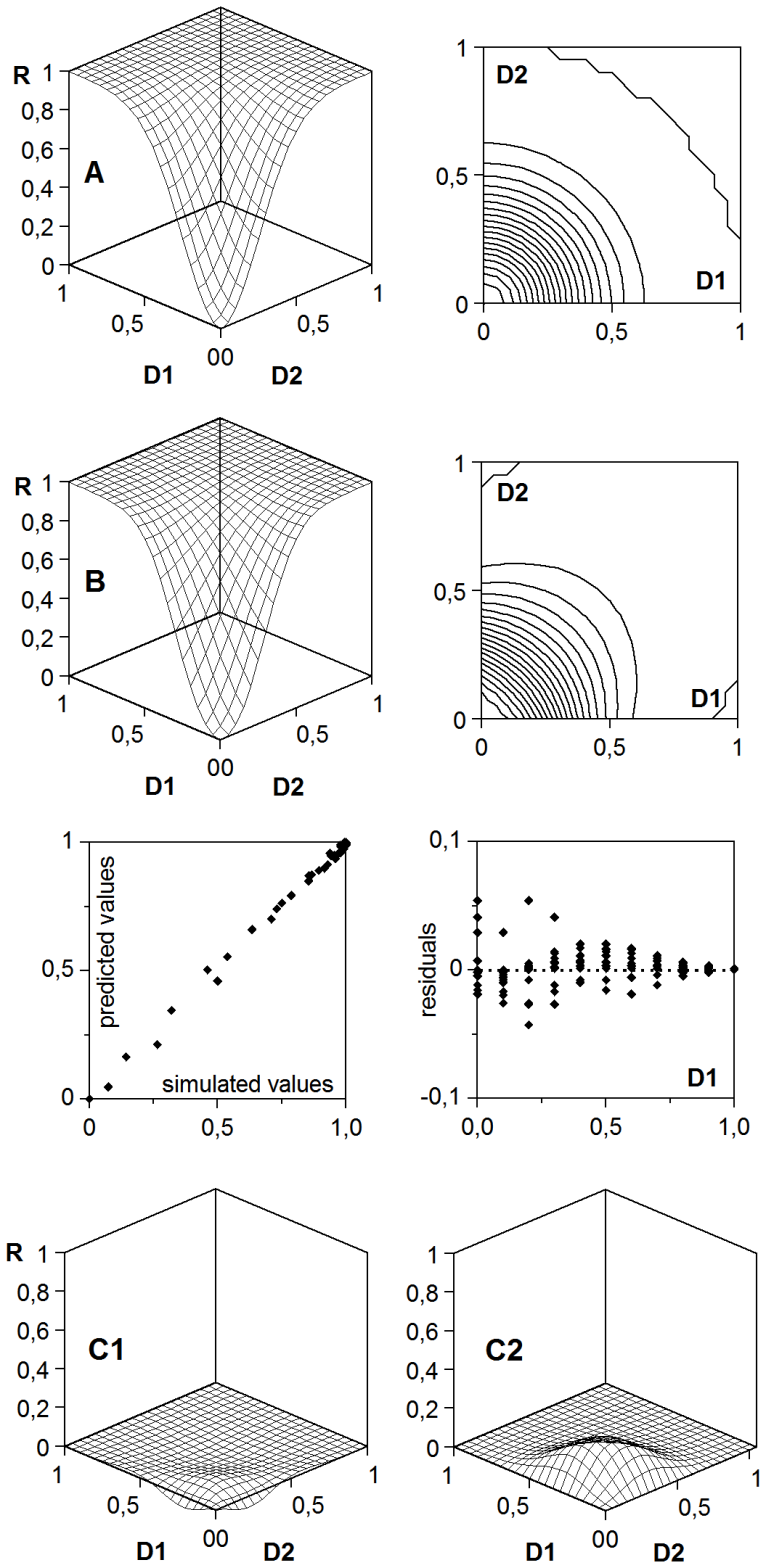
An example of ambiguous interpretation of a response through IA and CA hypothesis. A: IA response surface (defined by *K*_1_ = *K*_2_ = 1.00; *m*_1_ = *m*_2_ = 0.30; *a*_1_ = *a*_2_ = 2.00). B: result obtained when this surface is interpreted, through CA hypothesis, as a case of symmetrical antagonism (*K* = 1.00; *m* = 0.316; *a* = 2.348; *c*_D2_ = *c*_D1_ = 1.311. All coefficients statistically significant, with α = 0.05). Notice the lack of residual randomness. C: differences between CA minus IA (C1) and CA null interaction minus CA antagonism (C2) responses.

Notations as in Table 4. See also Figure 9 and Figure 10.

(^a^) apparent parameter (see text).

As a consequence of the equation (27), the CA hypothesis can be accepted when the individual responses differ in their *m* parameters and –given the relation (8)– in their maximum slopes, but should be rejected when such responses differ in their *K* parameters. This is consistent with the isobole approach: if the asymptotes differ, expressions as (4), (5) or (6) could only be applied to lower responses than the lowest asymptote, since the inverse function (9) only exists if *R*<*K*.

When interactions are included, the key notion of concentration addition should be preserved in any modification of the (27), what means that the doses should act as an additive block in a function with a single set of sigmoidal parameters (*K*, *m*, *a*). These conditions enable the cases that are described next.

#### 4.1. Effectors with different potency

This case was simulated by using the rule R5a and multiplying one of the doses by a *tox* factor (*tox* = 1 for effectors with equal potency). Results were fitted –with coded doses in the same interval– to the equation:
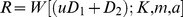
(28)which provided a precise description and produced straight isoboles with different intersection points on the doses axis (Figure 8). The *u* coefficient (*u*>1 if the first effector has more potency than the second one) means that if a joint response is described by the equation (28), the *m*_2_ parameter of the individual response to the second effector is *m*_2_ = *m*×*u*.

#### 4.2. Synergy and antagonism

To obtain surfaces with isoboles like those associated with synergy and antagonism, the rule R5a must include the condition that an effector alters, unidirectional or reciprocally, the effective dose of the other one. By using π_Di_ terms like those π_θi_ defined by equation (22), the corresponding simulations (Figure 8 and Figure 9) were described by means of:
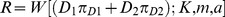
(29)


Contrary to the case of the perturbations, the equivalent dual solution based in the variation of the *m* parameter is not possible here.

#### 4.3. Interactions effector-receptor (α) and effector-threshold (λ)

If it is admitted that an effector E_1_ alters the number of active receptors α_2_ or the threshold λ_2_ of the effector E_2_, the conservation of the CA hypothesis requires to admit also that E_1_ alters the α_1_ or λ_1_ values. What in turn means to admit autoinhibitory or autocalytic effects. In fact, when this type of interactions are included in the rules R5, the response surfaces from the resulting simulations are limited by individual responses that decrease after a maximum or increase not asymptotically. Moreover, the estimates of the sigmoidal parameters from the joint response are only apparent (such as the Michaelian parameters in the presence of inhibitors), useful to predict the response surface, but without direct physical meaning in connection with individual responses, whose real parameters should be separately calculated using the model (7).

Avoiding now to discuss the realism of these behaviours, it can be pointed out that the response to lactic acid of some lactic acid bacteria seems to illustrate them, at least in their autoinhibitory modality [35]–[37].. Thus, the response to this acid of *Leuconostoc mesenteroides* [38] showed a profile –like that resulting from an enzymatic kinetics under substrate inhibition– which was described by including in the logistic equation a dose-depending term depressing the asymptotic value. But the description is also feasible –and more accurate– using a modified Weibull model as: 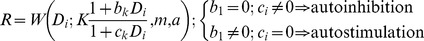
(30)


In any case, these α and λ interactions under IA response can be described, in their most complex forms, by modifying the *K* and *m* parameters with interaction terms:
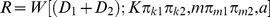
(31)


Although these effects increase or decrease the response with respect to that expected in null interaction, the isoboles differ markedly (Figure 9) from those produced by the equation (29) and corresponding to synergy and antagonism.

As in IA mode, all the possible options under CA hypothesis can be unified in a generalized model which, in its most complex form –again in practice simpler cases with several π_θi_ = 1 should be expected– can be formulates as:
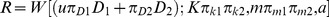
(32)


### 5. Broad and strict sense of the notions of synergy and antagonism

The frequent and not always enlightening discussions about synergy and antagonism have led some authors to consider synergy as an «ineffective mixture risk definition» [10]. However, we believe that both concepts are important, and that their confused sides can be debugged by suppressing some common, not justified assumptions.

Firstly, their formal aspects should be distinguished from the factual ones. From a factual perspective, as it has been said, synergy and antagonism are the result of interactions that increase or decrease the response with respect to that expected in null interaction. This is a broad and unambiguous definition, but it requires taking into account the following issues:

1. Synergistic and antagonistic responses can be generated by any of the interactions (D, α or λ) considered in preceding sections. This implies that such responses can have diverse origins, that these origins require different formal descriptions, and that these descriptions are dependent not only on the elements involved in a given interaction, but also on the inherent mechanism of the system under null interaction conditions.
2. Association between concave/convex isoboles and synergy/antagonism is limited and misleading. In IA mode, as it has been seen, it lacks sense, and in CA mode it lacks general validity. As an example, the reciprocal synergistic α-interaction (Figure 9) increases the response in the entire domain with respect to null interaction, in spite of which its isoboles are straight. In fact, that association is only applicable to D-interactions in CA mode, where the corresponding regular series of concave/convex isoboles enable clear contrasts with the straight series which are typical –although no exclusive– of the null interaction.
3. If the usual isobolographic convention in CA framework is followed, synergy/antagonism could be defined in a strict sense as those effector-effector interactions that increase/decrease the response. Such a definition, however, would exclude arbitrarily other interactions (as α or λ ones) with similar net effects.
4. Unfortunately, satisfactory expedients for typifying synergy/antagonism by means of a single value or a particular isobole do not exist, since the differences between these conditions and null interaction vary throughout the corresponding response surfaces (Figure 10C2).
5. Furthermore, there are not theoretical reasons which prevent interactions with opposite regional effects on the response surface (*e.g*. E_1_ increases the effective dose of E_2_, and E_2_ increases the parameter *m* of the response to E_1_). Consequently, synergy and antagonism can be simultaneously detected in different regions throughout a given response surface.

### 6. More insufficiencies and ambiguities of the IA-CA dualism

The frequent inconclusive character of IA and CA hypothesis is a well-documented experimental fact [39], whose justification was suggested in theoretical section 3. A different justification is provided by the simulations described *Simulation of perturbations of the response to an effector* and *Partial forms of independent action* sections. Indeed, when the conditions that define the IA hypothesis are altered in biologically plausible forms, response surfaces with cooperative or competitive effects are obtained, which cannot be acceptably described by any of the possibilities of the IA-CA scheme.

An additional cause of ambiguity –difficult to detect in the absence of explicit models– derives from a more formal issue. The IA and CA response surfaces are in general clearly distinguishable when the asymptotes of the individual responses are less than 1, as in the examples of Figure 6, Figure 7, Figure 8 and Figure 9. But the distinction turns more problematic as these asymptotes move closer to 1, since in such a case the IA surface losses its peculiar top region, in which the joint response surpasses the asymptotes of the individual responses. Figure 10 shows an IA surface obtained by assigning arbitrary parametric values (with *K*_1_ = *K*_2_ = 1) to the model (1), which could be significantly typified as a case of antagonism in CA mode by any assessment method, especially if the results are “blurred” by the experimental error. Similarly, a reciprocal asymmetrical synergy in IA mode is practically indistinguishable from synergy in CA mode. In such cases, the false hypothesis could only be detected by the lack of randomness of the residuals, if the number of observations is sufficient and the experimental error is reasonable.

All these reasons lead to doubt about the generalization to more than two effectors of any IA-CA discrimination method, since the probability of all kind of ambiguities increases with the number of agents considered. The main justification of this generalization is the experimental economy in the research of the joint effect of many effectors at moderate levels, an important issue in environmental toxicology, which seems practically unapproachable through the assay of binary combinations. However, it seems as well that the simplification of the experimental arrays only will produce even more ambiguous results.

### Experimental examples

Some elements of the approach proposed here are exemplified by two above mentioned cases of study: the larval growth inhibition in sea urchin by the joint action of hydrocarbons and dispersants [16], and the inhibitory perturbation by ouabain of the haemolytic action of palytoxin [27]. In fact, the need to clarify problems as those arose in these cases was the origin of the simulation-based systematics we have attempted in this work.

We present now an experimental example in connection with the antioxidant activity, a field in which the possible interactions between both natural and synthetic products are formally equivalent to the toxicological ones, and they raise similar discussions. The example refers to the inhibition of crocin oxidation by the joint action of two well-known antioxidants: ascorbic acid and trolox. Triplicate analytical results were obtained by monitoring of the oxidation kinetics in 8×8 concentration arrays, using a microplate method [40], [41]. Inhibitory responses were quantified through the ratio between areas under kinetic profiles at the end point of the control [40], [42]–[44].

Antioxidants can compete with the oxidizable substrate for oxygen or the source of radicals (primary antioxidants), or for radicalized products that are formed in more advanced oxidation phases (secondary antioxidants). Therefore, their modes of action are in agreement with the diagrams of Figure 2 and they can be analysed using the general equations [31] or [37].

**Figure 11:**
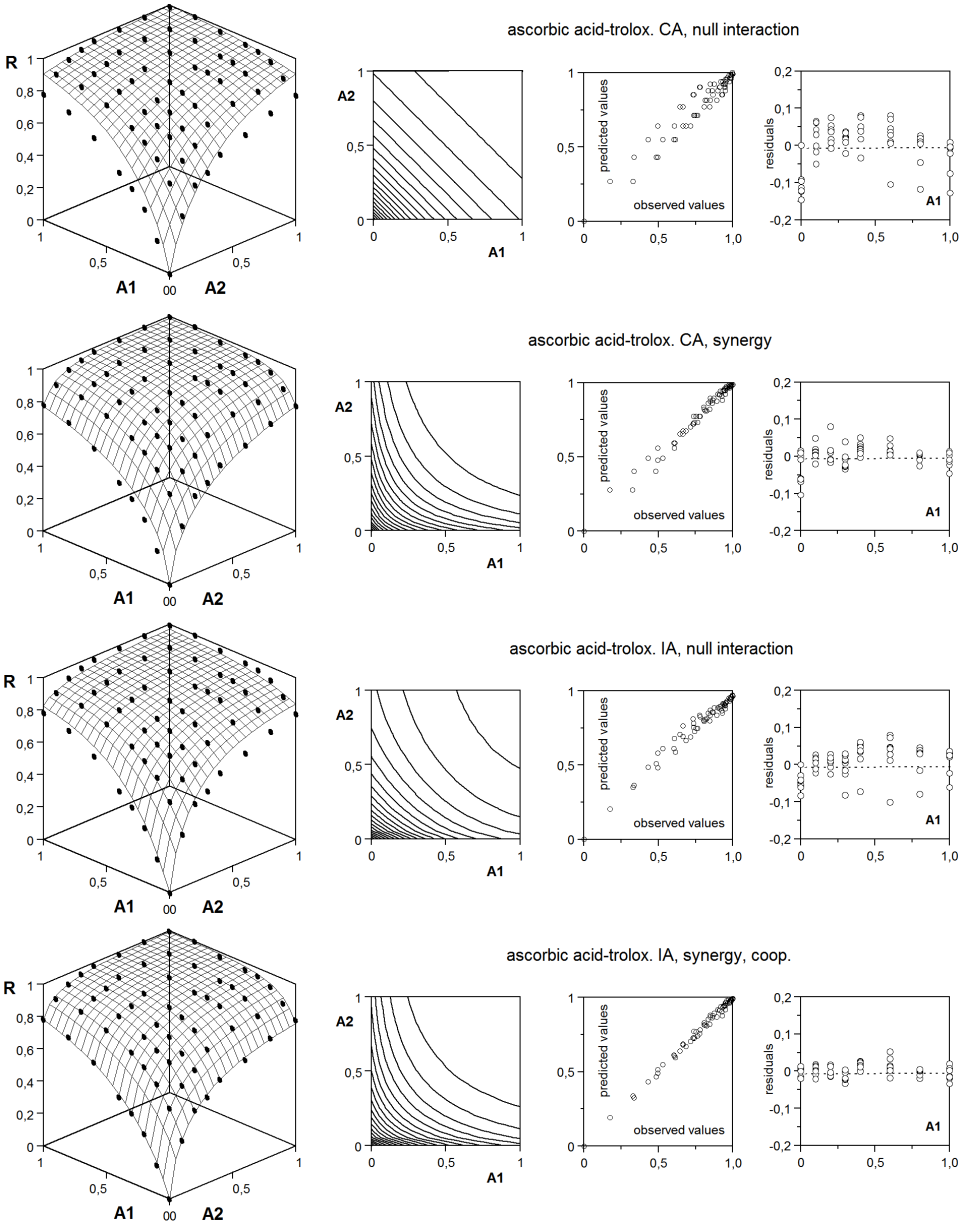
Joint effect of ascorbic acid (A_1_) and trolox (A_2_) on crocin oxidation under different hypotheses. Keys and graphic criteria as in Figure 6, with parametrtic variations replaced by residuals, which are more informative in this case. See details in text, and numerical results in.

In this regard, the example of ascorbic acid and trolox is interesting, because it shows some of the features in which a clear decision is difficult (Figure 11 and Table 6). Although null interaction cannot be accepted under both IA and CA hypotheses, when a synergistic effect is admitted, the difference between the two (statistically significant) options becomes small. Residual distribution inclines the decision towards IA, but a more accurate characterization is a *predominantly* independent action, with synergy and a cooperative unspecific effect. On the other hand, it should be pointed out that a conventional analysis (use of a model (1) for IA hypothesis and a contrast on the 50% isobole for CA hypothesis) would lead to decide a CA

**Table 6:** Antioxidant joint action of ascorbic acid (A_1_) and trolox (A_2_) on crocin oxidation.

independent action				concentration addition			
		null interaction	synergy			null interaction	synergy
response	K_1_	0,880±0.110	0,830±0.093	joint	K	1.021±0.074	0,988±0.019
to A_1_	m_1_	0,150±0.038	0,154±0.039	response	m	0.263±0.038	0,304±0.024
	a_1_	0,777±0.232	0,667±0.124		a	0.853±0.164	0,663±0.063
response	K_2_	0,883±0.146	0,922±0.084	relative potency	u	-	-
to A_2_	m_2_	0,260±0.073	0,332±0.057	A_1_ altering	b_2D_	-	-
	a_2_	1,009±0.286	0,909±0.099	eff. conc. of A_2_	c_2D_	-	-
A_1_ as perturbing	b_2k_	-	-	A_2_ altering	b_1D_	-	-
factor for	c_2k_	-	-	eff. conc. of A_1_	c_1D_	-	8.227±2.235
params. of the	b_2m_	-	-	A_1_ as perturbing	b_2k_	-	-
response to A_2_	c_2m_	-	2.619±0.892	factor for	c_2k_	-	-
A_2_ as perturbing	b_1k_	-	-	params. of the	b_2m_	-	-
factor for	c_1k_	-	-	joint response	c_2m_	-	-
params. of the	b_1m_	-	-	A_2_ as perturbing	b_1k_	-	-
response to A_1_	c_1m_	-	-	factor for	c_1k_	-	-
comp/coop	s	-	0.980±0.038	params. of the	b_1m_	-	-
	adj. r^2^	0.951	0.9932	joint response	c_1m_	-	-
					adj. r^2^	0.9195	0.9811
res.sk.		−0.827	0.335	res.sk.		−0.969	−0.677

Hypotheses of null interaction and synergy are compared, under the suppositions of independent action and concentration addition, through the fitting of the experimental results to the respective generalized models. Adj. r^2^: coefficient of multiple determination; res. sk.: residual skewness. See Figure 11 and text for details.

## Discussion

The approach we have defended here is supported on three statements: s1) in any algebraic macroscopic model, any interaction between effectors is necessarily translated into –reciprocal or not– modifications of the parametric values describing the individual action of each effector. In our modelling, such modifications can adopt increasing or decreasing forms, with constant, increasing or decreasing slope in each case; s2) the algorithms used for describing microscopic facts underlying common chemical and biological interactions produce realistic simulations as long as any hypothetical mechanism considered can be: a) defined in terms of levels of effectors, enhancers, inhibitors, receptors and response thresholds; b) expressed involving these elements in Boolean propositions; s3) macroscopic consequences of all the microscopic mechanisms considered were satisfactorily described by the proposed macroscopic modelling.

With these premises, two complementary queries emerge. The first concerns the degree in which the microscopic interactions considered are important for defining observable consequences as synergy or antagonism. The second one refers to the possibility that a concrete microscopic mechanism can be specifically identified through the macroscopic model obtained from a given data set.

Regarding the first query, it can be underlined that the two suppositions in which our approach would be insufficient are: 1) parametric variations (with discontinuities, singularities, and very pronounced inflection points) which cannot be described according to s1; 2) mechanisms which cannot be reduced, in the last analysis, to the terms s2. Since none of these suppositions are too plausible, it can be accepted that the considered interactions are considerably relevant –an absolute answer seems impossible in a factual science–, and that, in any case, our double approach will help in the search for alternative types of interactions.

The second query leads to admit that our modelling is mechanistically in the microscopic-macroscopic direction, but phenomenological in the opposite one. Therefore, it obeys the general fact that none mechanism can be unequivocally deduced from any macroscopic description, because different mechanisms can generate the same phenomenal profile. In perturbator-effector interactions, a reasonably mechanistic systematics is possible on the basis of the relationships between parametric variations (Table 2). But in effector-effector interactions, these relations provide only mechanistic suggestions, whose validation requires additional experiments as those applied in enzymatic kinetics to the identification of the different inhibition modalities.

Nevertheless, our modelling produces explicit algebraic equations able to describe accurately a set of situations more diverse and realistic than those considered in other alternatives, and allows to classify (at least according to the parametric variations) different modalities of synergy and antagonism. The proposed approach solves some recalcitrant and controversial aspects of these concepts, as well as the necessary distinction between the factual and formal sides of these phenomena, and it exposes several types of theoretical reasons that explain the abundance of experimental results that are inconclusive in the IA-CA framework, as it was pointed out by other authors [11], [12]. These reasons have perhaps diverse orgin, but they are related, firstly, with the fact that IA and CA hypotheses are far from elemental statements. As it is proven by the rules R1 to R4, IA hypothesis involves conditions that can be combined in ways that do not obey any of the two modes of action, but whose macroscopic consequences can be satisfactorily described in the frame of our proposal.

## Supporting Information

Figure S1 Simple radial (A), concentric radial (B), equiadditive (C) and complete (D) designs.
(TIF)

Supporting Information S1 Experimental design.
(DOC)
