# Supplementary figures and images for: Dose-Response Analysis in the Joint Action of Two Effectors. A New Approach to Simulation, Identification and Modelling of Some Basic Interactions
Source: PLoS One. 2013 Apr 24;8(4):e61391. doi: 10.1371/journal.pone.0061391 (PMC3634793; doi:10.1371/journal.pone.0061391)

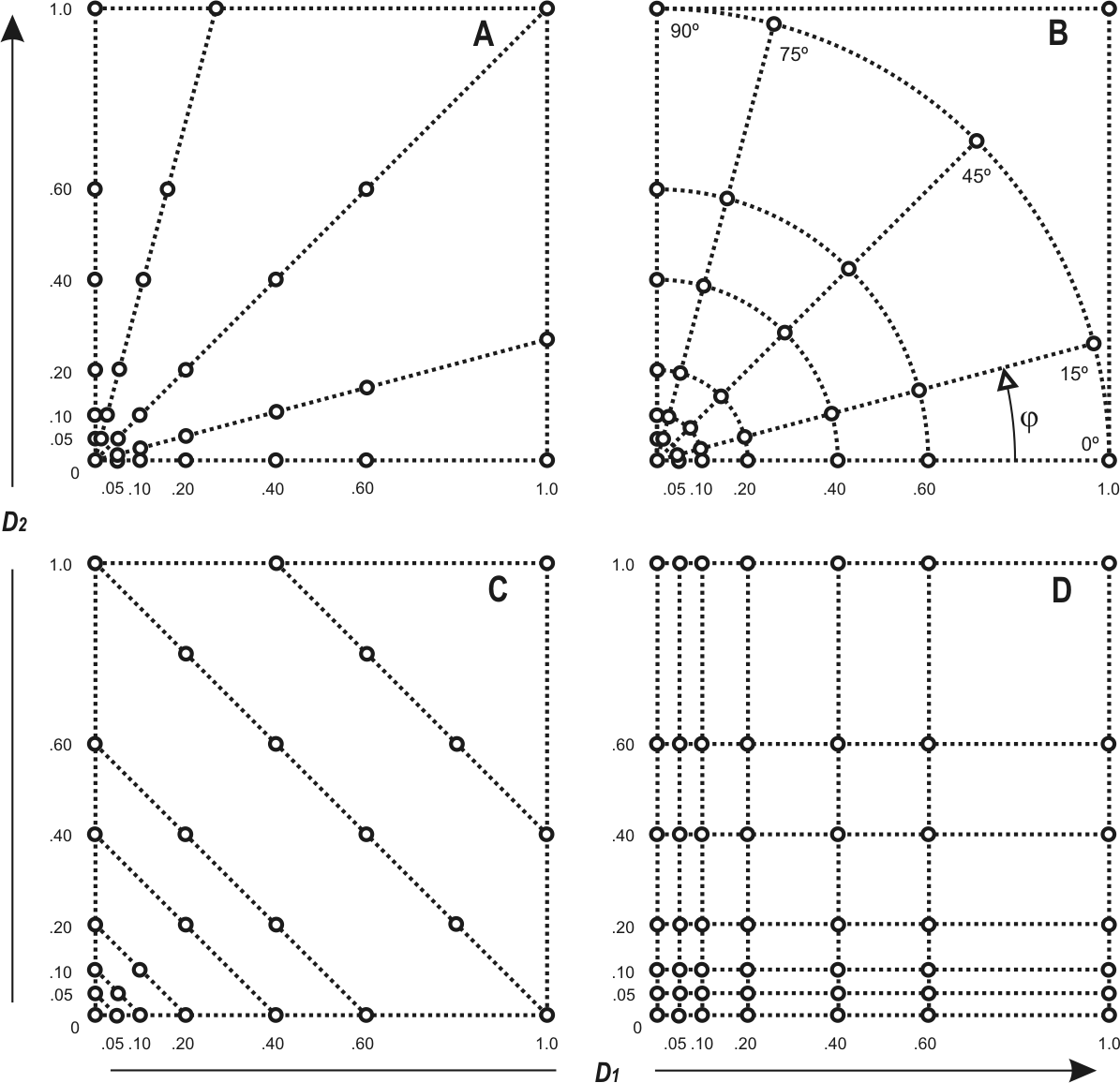

Supplement: Figure S1 — Simple radial (A), concentric radial (B), equiadditive (C) and complete (D) designs. (TIF) [file pone.0061391.s001.tif]
