# Supplementary material for: Dose-Response Analysis in the Joint Action of Two Effectors. A New Approach to Simulation, Identification and Modelling of Some Basic Interactions
Source: PLoS One. 2013 Apr 24;8(4):e61391. doi: 10.1371/journal.pone.0061391 (PMC3634793; doi:10.1371/journal.pone.0061391)
Supplement: Supporting Information S1 — Experimental design. (DOC) [file pone.0061391.s002.doc]

SUPPORTING INFORMATION

Experimental design

In any design, a convenient practice is to code the doses (dividing them by the maximum ones) in such a way that both individual series include the same values (*D*_i_) within the [0, 1] interval. Together with the encoding of the response in the same interval, this facilitates the fitting process and provides standardized parametric estimates. Once the *D*_i_ series is defined, there are several reasonable modes to establish the mixed doses covering the experimental domain (figure SI.1).

*Simple radial design*: besides the individual series *D*_1i_, 0 and *D*_2i_, 0 (*D*_1i_=*D*_2i_=*D*_i_), this option includes several additional sets of mixed doses (*d*_1i_, *d*_2i_), each set defined by a constant ratio (*d*_1i_/*d*_2i_=*Q*) between the concentrations of both effectors. Thus, the mixed dose set located along the radius defined by *Q*_n_ is:

If *Q*_n_ ≤ 1: $d_{1i\left[Q_{n}\right]}=D_{i}$ ; $d_{2i\left[Q_{n}\right]}=D_{i}\times Q_{n}$

If *Q*_n_ > 1: ; $d_{2i\left[Q_{n}\right]}=D_{i}$

*Concentric radial design*: as the preceding one, but with mixed doses defined from the angle (ϕ_n_) that each radius makes with the variable representing the *D*_1i_ series:

$d_{\text{1i}\left[\phi_{\text{n}}\right]}\text{=}D_{i}\times\text{cos}\phi_{\text{n}}$ ; $d_{\text{2i}\left[\phi_{\text{n}}\right]}\text{=}D_{i}\times\sin\phi_{\text{n}}$

Number of radii and values of ϕ_j_ (or *Q*) can be freely fixed, taking into account that high (~75º) and low (~15º) values of ϕ_j_ favour the detection of interactions.

*Equiadditive design*: mixed doses are grouped in series defined by a constant sum (*d*_1i_+*d*_2i_=*S*). Thus, *v* being the desired number of doses per series:

If *S*_n_ ≤ 1: $d_{1i\left[S_{n}\right]}=S_{n}-h_{v}\left(\frac{S_{n}}{v-1}\right)$ ; $d_{2i\left[S_{n}\right]}=S_{n}-d_{1i\left[S_{n}\right]}$ ; (*h*_v_=0, 1,…*v*–1)

If *S*_n_ > 1: $d_{1i\left[S_{n}\right]}=\left(S_{n}-1\right)+h_{v}\left(\frac{1-S_{n}+1}{v-1}\right)$ ; $d_{2i\left[S_{n}\right]}=S_{n}-d_{1i\left[S_{n}\right]}$ ; (*h*_v_=0, 1,…*v*–1)

*Radial equiadditive design*: mixed doses fulfil simultaneously the conditions *d*_1i_/*d*_2i_=*Q*_n_ and *d*_1i_+*d*_2i_=*S*_n_, therefore:

$d_{1i}=S_{n}/\left(1+Q_{n}\right)$ ; $d_{2i}=S_{n}\left[1-\left(1/\left(1+Q_{n}\right)\right)\right]$

*Complete design*: is the most intuitive experimental plan, combining simply all the doses of an effector with all doses of the other.

In principle, each design offers specific advantages for identifying concrete modes of action and interaction by comparing, through an appropriate statistical criterion, the observed responses at certain dose series with the expected ones under IA or CA null interaction hypotheses. However, both the simulations with logical rules and those based on the respective explicit models prove that the grounds of this supposition are weak, and its results doubtful. Indeed, as it was discussed in 3.5 and 3.6, the response surface properties in joint actions imply: 1) numerous indistinguishable situations as analysed by means of radial or equiadditive series; 2) responses whose behaviour in a given region of the experimental domain does not represent necessarily what takes place in other regions.

In fact, the most discriminative resort is the explicit model, and, for obtaining it, the complete design is the most advisable. Even if one wants to disregard doubtful auxiliary functions (see below), the responses to a same dose set of an effector in the presence of increasing doses of the another form very specific systematic sequences. These sequences are more informative than radial or equiadditive ones, and can be advantageously subjected to the comparative criteria above mentioned, by using equations (24) and (27), or the responses generated with the equations (24) to (31). Additionally, the bootstrap method proves that a good coverage of the experimental domain (complete design) is more efficient than an increase of the number of replicates to minimize the effects of the experimental error.
